# Single-gap superconductivity in Mo_8_Ga_41_

**DOI:** 10.1038/s41598-019-49846-y

**Published:** 2019-09-19

**Authors:** Miroslav Marcin, Jozef Kačmarčík, Zuzana Pribulová, Michal Kopčík, Pavol Szabó, Ondrej Šofranko, Tomáš Samuely, Viliam Vaňo, Christophe Marcenat, Valeriy Yu. Verchenko, Andrei V. Shevelkov, Peter Samuely

**Affiliations:** 1 Centre of Low Temperature Physics, Institute of Experimental Physics SAS, and Faculty of Science, P. J. Šafárik University, 040 01 Košice, Slovakia; 2 Faculty of Electrical Engineering and Informatics, Department of Physics, Technical University, SK-04001 Košice, Slovakia; 3 SPSMS, UMR-E9001, CEA-INAC/UJF-Grenoble 1, 38054 Grenoble, France; 4 Department of Chemistry, Lomonosov Moscow State University, 119991 Moscow, Russia; 5 National Institute of Chemical Physics and Biophysics, 12618 Tallinn, Estonia

**Keywords:** Superconducting properties and materials, Phase transitions and critical phenomena, Electronic properties and materials

## Abstract

In this paper, the potential existence of two-gap superconductivity in Mo_8_Ga_41_ is addressed in detail by means of thermodynamic and spectroscopic measurements. A combination of highly sensitive bulk and surface probes, specifically ac-calorimetry and scanning tunneling spectroscopy (STS), are utilized on the same piece of crystal and reveal the presence of only one intrinsic gap in the system featuring strong electron-phonon coupling. Minute traces of additional superconducting phases detected by STS and also in the heat capacity measured in high magnetic fields on a high-quality and seemingly single-phase crystal might mimic the multigap superconductivity of Mo_8_Ga_41_ suggested recently in several studies.

## Introduction

Two-gap superconductivity is a compelling phenomenon as it comprises new riches of condensed matter physics, including, for example, a new mechanism for spontaneous symmetry breaking in systems of superconducting vortices^1^ or the existence of fractional vortices^2^. The hunt for additional examples continues since the experimental justification of two energy scales was established in MgB_2_^3^ in 2001.

Two-gap superconductors are characterized by the existence of two distinct energy gaps that reside on separated parts of the Fermi surface interconnected by some interband scattering. This scattering results in the closing of both energy gaps at the same critical temperature *T*_*c*_. Techniques like scanning tunneling spectroscopy (STS) are able to show two gaps directly, but other measurements sensitive to quasiparticle density of states (DOS) can be useful, too. For example, the temperature dependence of the heat capacity^4^ and of the upper critical magnetic field *H*_*c*2_^5^ can be used to consistently model two gaps in the system. For more information about different aspects of the two-gap superconducitvity see e.g. Special issue of Physica C: Recent Advances in MgB_2_ Research^6^. Unfortunately, a presence of additional phase(s) of small volume may sometimes exhibit similar behavior mimicking a true multi-gap case. Thus, prior to reaching a definitive conclusion about existence of multiple order parameters, a combination of techniques, which address several aspects of the phenomenon, are required to establish a consistent description of the underlying superconducting state.

Recently, it was suggested Mo_8_Ga_41_ features two-gap superconductivity^7–9^ that vanishes upon V for Mo substitution^7^. The material is a member of endohedral gallium cluster compounds. In the structure of Mo_8_Ga_41_, each Mo atom is placed inside a cage of 10 Ga atoms forming endohedral clusters that share all their vertices. This architecture resembles that of perovskite oxides, among which various important superconductors, including high-*T*_*c*_ oxides, can be found. Recently, it was also noted that superconductivity and structural stability probably compete in this family of galium-based superconductors^10^. Therefore, unconventional features in Mo_8_Ga_41_ might be anticipated. There are several members of this family that are known to be superconducting. Besides Mo_8_Ga_41_ with *T*_*c*_ ~ 9.8 K^11^, superconductivity was also reported in Mo_6_Ga_31_ with *T*_*c*_ ~ 8 K^12^.

The superconducting properties of Mo_8_Ga_41_ were studied by transport and thermodynamic measurements in ref.^13^. Heat capacity and magnetic susceptibility were measured on a collection of single crystals glued together in order to obtain reasonable signal. For transport measurements, polycrystalline samples were used. Indications of the strong-coupling superconductivity in the system were found. In a subsequent study by means of muon spin rotation/relaxation spectroscopy, it was suggested^7^ that two superconducting energy gaps exist in Mo_8_Ga_41_. Later, reports on possible existence of two energy gaps in Mo_8_Ga_41_ followed from the critical current^8^ and STS measurements^9^.

Here, we present a comprehensive study of the superconductivity in individual, tiny single-crystals of Mo_8_Ga_41_ investigated by bulk and surface sensitive methods. Specifically, ac-calorimetry is employed to study fine structure of the heat capacity anomaly at the superconducting transition by sweeping temperature or magnetic field, while scanning tunneling microscopy (STM) and STS are used to directly probe the superconducting gaps. Our analysis based on the heat capacity measurements shows that the system is clearly a single s-wave gap superconductor, however, traces of other minor superconducting phases may mimic the multigap behavior in the surface-sensitive techniques as evidenced by the STS data.

## Experiment

Single crystals of Mo_8_Ga_41_ were synthesized using the flux growth method. Details of synthesis can be found in the previous report^13^. The obtained crystals were characterized by a combination of electron probe x-ray microanalysis and single-crystal x-ray diffraction, and no deviations from the Mo_8_Ga_41_ composition and crystal structure were found. The residual-resistance-ratio of ${\rm{RRR}}=15.4$ found in the standard electrical transport measurements indicates good quality of the crystals.

Thermodynamic properties were measured by the ac-calorimetry^14^ when periodically modulated sinusoidal power is applied and resulting sinusoidal temperature response is measured. In our case, a light emitting diode was used as a contact-less heater with an optical fiber to guide the heating power towards the sample. Individual crystals were glued on a chromel-constantan thermocouple, which served both as a sample holder and a thermometer to detect oscillations of the sample temperature. The thermocouple was placed on a copper block that served as a thermal bath and was regulated by a resistive heater and Cernox thermometer. A precise *in situ* calibration of the thermocouple in magnetic field was obtained from measurements on ultrapure silicon. The magnetoresistance of the Cernox thermometer has been thoroughly calibrated in field, from 0.2 K to 4 K against Ge sensor placed in a compensated area of superconducting magnet and between 2 K and 20 K against capacitor. The corrections of the Cernox and the thermocouple in magnetic field were included in the data treatment. In order to subtract the addenda from the total heat capacity, the empty thermocouple was measured in zero magnetic field and in fields up to 10 T. In the experiment, the heat was supplied to the sample at a frequency of several Hertz. Measurements were performed down to 600 mK in ^3^He cryostat in 8 T horizontal, and 10 T vertical magnets. The magnetic field was applied both perpendicular and parallel to the flat sample facet attached to the thermocouple. Since we did not find any significant difference in a position of the superconducting transition for the two magnetic field orientations (the difference was less than 1%), we consider system to be isotropic.

Superconducting properties of the sample surface were probed by STM. To avoid any contamination prior to the experimental study, the surface was treated either ex situ by polishing on an Al_2_O_3_ plate or in situ by mild Ar^+^ sputtering in the Specs UHV STM system. Subsequent STM and STS measurements performed at the base temperature of 2 K showed no noticeable difference between the two surface treatment methods. Therefore, further STM and STS experiments were performed on the ex situ treated samples employing our homemade STM system immersed in a Janis SSV ^3^He cryostat allowing measurements down to 400 mK in magnetic fields up to 8 T. A gold STM tip was used for measurements. The spectroscopy measurements were performed by obtaining current-voltage (*I* − *V*) characteristics, then numerically differentiating the *I* − *V* curves to acquire the tunneling conductance spectra *G*(*V*) = d*I*(*V*)/d*V* and normalizing those to the normal-state conductance *G*_*N*_. Tunneling spectra were fitted by the tunneling conductivity model for the normal metal-superconductor (N-I-S) tunneling junction^15^, with the thermally smeared BCS density of states of the superconducting electrode. Two-gap spectra were tested by fitting the data by the convolution of two BCS conductance spectra, assuming two different energy gaps Δ_1_ and Δ_2_ with complementary weights *w*_1_ and *w*_2_ = 1 − *w*_1_, respectively. Spectral conductance maps were measured at *T* = 450 mK in zero magnetic field using the Current Imaging Tunneling Spectroscopy technique^16^ with the 128 × 128 spatial resolution for a given surface area and using a bias voltage range of ±8 mV.

## Results

**Figure 1:**
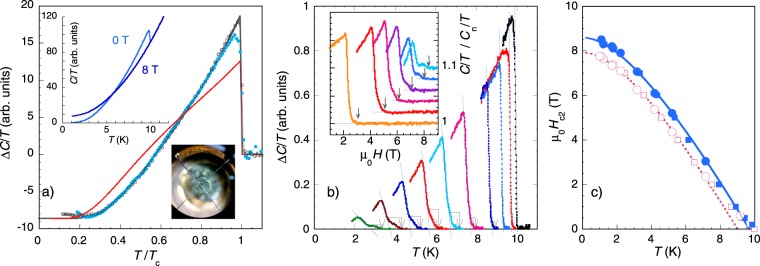
Heat capacity of an individual single crystal of Mo_8_Ga_41_: (**a**) Upper inset: heat capacity of the sample measured in 0 T and 8 T magnetic fields. Main panel: field-dependent part of the electronic heat capacity from the present study (empty black symbols), and from ref.^13^ (filled blue symbols), the grey line is the single-gap *α* model curve with 2Δ/k*T*_*c*_ = 4.4, the red line is the two-gap *α* model curve as described in the text. Lower inset: The sample mounted on a thermocouple with the optical fibre in the background. (**b**) Heat capacity after normal-state contribution subtraction. Blue lines are to highlight the main transition, broken arrows are to visualize the deviation from the main transition and point to the onset of superconductivity. Temperature sweeps measured in 7, 6, 5, 4, 3, 2, 1, 0.5, 0.2, and 0 T magnetic fields are shown from left to right. Inset: field sweeps of the heat capacity normalized to the normal-state contribution measured at 7.2, 5.2, 4.2, 3.2, 2.2, 1.7 K from left to right, for clarity not all measured curves are displayed and the plotted curves are shifted on Y-axis. (**c**) Upper critical field determined at the mid-point of the anomaly (empty symbols) and at the onset of the transition (filled symbols), from the temperature sweeps (squares) and field sweeps (circles), size of the symbols corresponds to the error bars. Lines are theoretical curves from the WHH model.

Figure 1 summarizes results obtained from the heat capacity measurements. The upper panel of Fig. 1a shows the total heat capacity divided by temperature *C*/*T* of an individual piece of single crystal of Mo_8_Ga_41_ measured in 0 T and 8 T magnetic fields. In zero magnetic field, an anomaly at the superconducting transition is clearly visible at ${T}_{c}\simeq 10\,{\rm{K}}$, and this feature is sharp, indicating the good quality of the sample. Contrastingly in the 8 T measurement, no clear anomaly is present. However, further analysis of the data showed that the superconductivity is not fully suppressed over the entire temperature range explored in this field, as an additional, small contribution is still present at low temperatures. For illustration, the lower inset of Fig. 1a shows a snap-shot of the experimental arrangement. The diameter of the fibre is 1 mm and the sample dimensions are approximately 550 × 250 *μ*m^2^ with the thickness of 150 *μ*m.

The main panel of Fig. 1a shows a plot of a field-dependent part of the electronic heat capacity from this study (empty black symbols), calculated as Δ*C*/*T* = *C*(0T)/*T* − *C*(8T)/*T*. Subtracting the 8 T data removes lattice contribution from the total heat capacity and leaves only the field-dependent electronic part. The main panel includes also the data from the previous report measured on a polycrystalline-like sample^13^ calculated in the same way (filled blue symbols). Overlap of the two sets of data is very good except at the transition, where the superconducting anomaly of single crystal is much sharper, and in the limited range at low temperatures. Note that not all of the data points from our present measurement (black symbols) are shown in the figure, only one point out of 50 is displayed for clarity. The solid grey line is a theoretical curve according to the *α*-model^17^ corresponding to a single s-wave gap superconductor with the coupling ratio of 2Δ/k*T*_*c*_ = 4.4. The theoretical line follows the experimental data in very good agreement, except the low-temperature region below 3 K, where the additional contribution to the heat capacity measured in 8 T is present as mentioned above. The value of 2Δ/k*T*_*c*_ = 4.4 exceeds the weak coupling limit of the BCS theory, indicating strong electron-phonon coupling in the superconducting state.

In Fig. 1b, evolution of the heat capacity with both temperature and magnetic field is shown. The main panel of the figure depicts superconducting anomaly while sweeping the temperature at fixed magnetic fields. At relatively low fields, the anomaly remains sharp, but when the field is increased, the anomaly broadens and splits in two in high magnetic fields. Arrows inserted in the figure highlight the onset of the transition. The inset of Fig. 1b shows several examples of measured and normalized heat capacity measurements while sweeping the magnetic field at fixed temperatures, the curves are shifted on Y-axis for clarity. Again, the arrows point to the onset of superconducting transition. Note that at low temperatures (two upper curves) the anomaly reveals two distinct jumps. This indicates that, even in the single crystal of Mo_8_Ga_41_, separate superconducting phases coexist. However, since the anomaly in zero magnetic field is very sharp, these superconducting phases most likely have identical critical temperatures, and they differ only slightly in their upper critical magnetic fields presumably related to a different local purity.

Using the temperature- and field-dependent heat capacity data, the upper critical field *μ*_0_*H*_*c*2_ as a function of temperature was constructed (Fig. 1c). Empty symbols refer to the mid-point of the anomaly, filled symbols correspond to the onset of superconducting transition marked by arrows in Fig. 1b; squares are determined from the temperature sweeps and circles from the field sweeps. Lines are predictions according to the Werthamer, Helfand and Hohenberg (WHH) model^18^ based on single s-wave energy gap, in the absence of paramagnetic and spin-orbit contributions ($\alpha =0$, ${\Delta }_{so}=0$) rescaled by different factors to match the low-temperature saturation of the *μ*_0_*H*_*c*2_ temperature dependence. The *μ*_0_*H*_*c*2_(*T*) dependence displayed by empty symbols (at the mid-point of the transition) reveals a pronounced positive curvature and deviates from the WHH theoretical curve above 7 K. The corresponding red WHH curve yields *T*_*c*_ = 9 K, which is significantly lower than the value observed in the heat capacity measurements in zero magnetic field. The positive curvature close to *T*_*c*_ of the *μ*_0_*H*_*c*2_ temperature dependence is a common consequence of the interplay between two gaps^5^ and might, at first glance, suggest two-gap behavior also in Mo_8_Ga_41_. However, the splitting of the superconducting transition observed in the heat capacity measurements suggests the presence of two phases, a dominant one and an additional one that appears with higher *μ*_0_*H*_*c*2_. This is probably due to shorter local mean free path leading to shorter coherence length and higher *μ*_0_*H*_*c*2_. Indeed, if *μ*_0_*H*_*c*2_ is determined at the onset of the transition (filled blue symbols in Fig. 1c) the theoretical WHH curve follows the experimental data in good agreement. The fact that the temperature dependence of *μ*_0_*H*_*c*2_ depends on a definition (midpoint, or onset of the superconducting transition) indicates the presence of tiny additionall superconducting phase in the same crystal. Here, we consider the onset of the transition to be a better criterion for the determination of *μ*_0_*H*_*c*2_.

**Figure 2:**
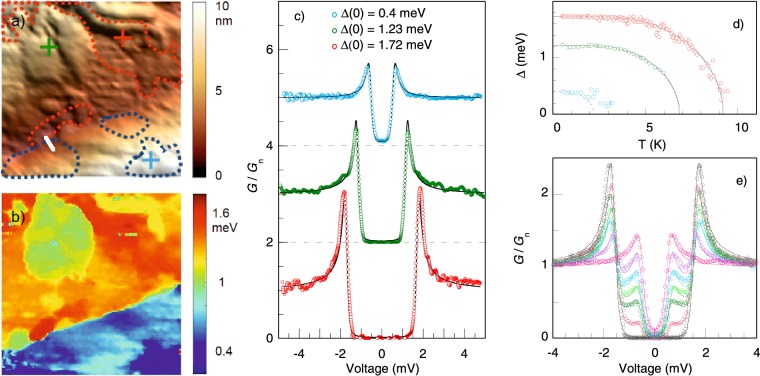
Tunneling microscopy and spectroscopy results: (**a**) Surface topography of a 200 × 200 nm^2^ surface area at 450 mK, the dashed lines are to highlight the areas with the largest (red) and the smallest (blue) value of the energy gap; (**b**) Superconducting gap map of the surface from (**a**); (**c**) Tunneling conductance spectra at 3 points of the scanned area marked by the crosses in (**a**) in matching colors, the curves are shifted for clarity. The dashed lines correspond to zero conductance level. The solid lines are theoretical fits; (**d**) Gap temperature dependences (symbols, size of which corresponds to the error bars) measured at the positions marked by the crosses in (**a**) and BCS fits (lines); (**e**) Tunneling spectra (symbols) measured along the white line in (**a**) between two well defined phases. Lines are theoretical fits involving the two contributions from both gaps.

Using STM we recorded multiple surface topographs and gap maps on different parts of the sample surface covering different areas (including areas as large as 700 × 700 nm^2^). Surface topography scans reveal inhomogeneous surface morphology consisting of a wide variety of differently shaped and sized regions, usually protruding 10–20 nm from the surface of the sample. This is accompanied by a wide spatial distribution of the energy gap Δ(0) as evidenced by tunneling spectra fits, suggesting the presence of several different surface phases. Figure 2 presents the results of topography measurements together with a spectral map measurement selected to illustrate this picture. The surface topography of a 200 × 200 nm^2^ area in Fig. 2a shows multiple regions, which become clearly distinguished on the gap map in Fig. 2b. The values of the superconducting gap Δ(0) range from 0.3 meV in the protruding area to 1.75 meV in the deepest parts of the valley in the middle of the scan. Figure 2c shows individual spectra of the three main distinct areas of the scan, measured at 450 mK after the scan was completed, with their positions marked by the crosses in Fig. 2a in the matching colors. The lines are theoretical fits giving the gap values of Δ(0) = 1.72, 1.23, and 0.4 meV from the bottom to the top. The temperature dependences of the energy gaps determined from three sets of spectra measured at those positions are depicted in Fig. 2d by symbols, the color code is kept the same. The lines represent a standard BCS behavior. For the two larger gaps, experimental data follow the theoretical curve in good agreement, yielding the values of *T*_*c*_ = 6.85, and 9.2 K. Together with the gap values, it leads to the strong superconducting coupling ratio of 2Δ/k*T*_*c*_ = 4.25 ± 0.1. The smallest energy gap in Fig. 2c closes at ~3 K, which is significantly low, yet higher than what would be expected for the strong coupling. The dashed line visualizes BCS curve with 2Δ/k*T*_*c*_ = 4.25. Superconducting energy gap survives up to higher temperatures, probably due to some proximity effect. Our measurements thus suggest that on the sample surface there are local areas with suppressed superconductivity. These areas are thicker than the coherence length of the sample ($\xi \sim 6.2\,{\rm{nm}}$), otherwise the proximity effect would lead to persistence of superconductivity in the junction up to the bulk *T*_*c*_.

In some specific cases, two-gap spectra with two pairs of coherence peaks were observed. They were found at the boundaries between the areas with different dominant gap values as demonstrated in Fig. 2e. The tunneling spectra measured along the white line in Fig. 2a exhibit a convolution of two separate spectra with different gap width (1.6 meV and 0.6 meV) as expected for the simultaneous tunneling to two neighboring phases. The relative contributions of the larger and the smaller gaps gradually shift, while moving along the line, starting from the larger single-gap spectrum on one side (black symbols in Fig. 2e), through two-gap spectra with progressivelly increasing weight of the smaller gap (*w*_1_ ~ 0.1; 0.35; 0.45; 0.6; 0.75), ending up with the smaller single-gap spectrum (pink symbols in Fig. 2e) on the other side of the line. The symbols in this figure are the experimental data and the lines are the sums of two densities of states with the above given energy gaps and their weights.

## Discussion

Our heat capacity and spectroscopic measurements are suitably described assuming a single energy gap. In some surface regions, especially in the deepest parts of the topography, the energy gap value was determined by STS to be ~1.75 meV and it was closed at the temperatures close to the bulk *T*_*c*_ value. This leads to the coupling ratio 2Δ/k*T*_*c*_ = 4.35, in accord with the heat capacity data. Thus this energy gap may be considered to be very close to the bulk one. The variety of other phases with suppressed superconductivity that were observed in our STS measurements are most probably related only to the surface since they are not reflected in bulk measurements, neither heat capacity nor X-ray diffraction. These bulk methods are not sensitive to inhomogeneities contribution of less than 1%. If we consider a 20 nm thick continuous surface layer of the sample with reduced critical temperature, the resulting volume of this layer would be below 0.1% of the total volume, and thus negligible in a scope of the bulk measurements.

Recently, Sirohi *et al*.^9^ reported STS spectra obtained at several positions on the surface of a Mo_8_Ga_41_ sample at 1.9 K, and these results were analyzed using a two-gap, rather than a single-gap, model. While the small energy gap varied between 0.8–1.1 meV, the large gap ranged between 1.45–1.68 meV, and the relative weight of the small gap possessed a wide variation, 0.2–0.77 depending on the position on the sample surface. Sirohi *et al*. also presented a temperature depedence of one spectrum with a larger gap of 1.45 meV and a critical temperature of 9 K, where both gaps close simultenously. This leads to the coupling ratio of 2Δ/k*T*_*c*_ = 3.74 for the larger gap, slightly above the BCS weak coupling limit of 3.52.

These results are not consistent with our heat capacity measurements that indicate the presence of strong coupling (2Δ/k*T*_*c*_ = 4.4) in the system. In addition, the overall temperature dependence of the heat capacity cannot be described consistently by taking into account the two gaps proposed in ref. ^9^. In order to visualize the difference, we show the solid red line in Fig. 1a that corresponds to the two-gap *α*-model fit with Δ_1_(0) = 1.63 meV, Δ_2_(0) = 1.1 meV, and the weight of the larger gap of 0.8. This set of parameters was selected as they result in the heat capacity behavior as close as possible to the observed one. Nevertheless, the two-gap model curve is clearly not consistent with our experimental data. Any other combination of the energy gap values proposed by Sirohi *et al*. leads to even more pronounced disagreement.

The surface inhomogeneities we have observed in STM/STS measurements may be related to the surface treatment since both polishing and argon sputtering may damage the surface. Another explanation would be the segregation and smearing out of Galium from the surface layer. The surface inhomogeneities may be also related to existence of several phases close to Mo_8_Ga_41_, and Mo_6_Ga_31_ is one example known to be superconducting below *T*_*c*_ = 8 K^12^. Actually, Mo_8_Ga_41_ and Mo_6_Ga_31_ can be combined into the Mo_*n*_Ga_5*n*+1_ family of superconductors. Although the $n=4$ member, Mo_4_Ga_21_, was not synthesized individually, it can be stabilized if gallium is partially replaced by a chalcogen. Indeed, Mo_4_Ga_21−*y*_S_*y*_, Mo_4_Ga_21−*y*_Se_*y*_, and Mo_4_Ga_21−*y*_Te_*y*_ compounds have been synthesized, and they show superconducting properties below *T*_*c*_ ~ 5 K in zero magnetic field^19^. On general, Mo_*n*_Ga_5*n*+1_ compounds exhibit a clear structural relationship as their structures are built by MoGa_10_ polyhedra and Ga_13_ cuboctahedra, which are centered by unique Ga atoms. Mo_4_Ga_21−*y*_Ch_*y*_ (Ch = S, Se, Te), Mo_6_Ga_31_, and Mo_8_Ga_41_ are individual compounds, which are superconductors below ~5, 8, and 10 K, respectively, representing different energy scales of the superconducting gaps. Their crystal structures are close to each other with the only slight difference lying in the way how MoGa_10_ polyhedra are packed and organized. Note also that these phases have very close compositions, which are ${{\rm{MoGa}}}_{5\tfrac{1}{4}}$, ${{\rm{MoGa}}}_{5\tfrac{1}{6}}$, and ${{\rm{MoGa}}}_{5\tfrac{1}{8}}$. Therefore, we assume that a single crystal of Mo_8_Ga_41_ may contain the surface domains, where the packing of MoGa_10_ polyhedra is slightly different resembling those in the Mo_*n*_Ga_5*n*+1_ series for *n* = 4, 6, and 8. The formation of such superconducting domains may be responsible for the appearance of distinct surface regions with different superconducting energy gaps.

## Conclusions

In summary, we have performed a detailed study of the superconductivity in Mo_8_Ga_41_ (*T*_*c*_ = 9.9 K) by means of bulk and surface sensitive techniques, i.e. ac-calorimetry and scanning tunneling microscopy/spectroscopy applied to the same single crystal. The heat capacity data is consistent with the single-gap *α* model with the coupling ratio of 2Δ/k*T*_*c*_ ~ 4.4 and clearly excludes the existence of the second energy gap in the system. The heat capacity anomaly at the superconducting transition splits in high magnetic fields, providing evidence of a minor additional superconducting phase with the same *T*_*c*_ but a different *H*_*c*2_. If the phase with larger upper critical magnetic field is taken into account, the positive curvature of *μ*_0_*H*_*c*2_ temperature dependence close to *T*_*c*_ diminishes leading to good agreement of *μ*_0_*H*_*c*2_(*T*) with the WHH model. The presence of multiple superconducting phases, this time with different *T*_*c*_ values, is clearly visible on the surface of the studied sample, where our local STM/STS measurements reveal broad distribution of the superconducting energy gaps. The largest of the observed energy gaps is consistent with the heat capacity data. Thus we can conclude that there is only one intrinsic superconducting energy gap in Mo_8_Ga_41_.

## Comments

The datasets generated during and/or analysed during the current study are available from the corresponding author on reasonable request.
